# Sex differences in atrial fibrillation in India: Insights from the Kerala‐AF registry

**DOI:** 10.1002/joa3.13195

**Published:** 2024-12-01

**Authors:** Peter Calvert, Yang Chen, Ying Gue, Dhiraj Gupta, Jinbert Lordson Azariah, A. George Koshy, Geevar Zachariah, K. U. Natarajan, Gregory Y. H. Lip, Bahuleyan Charantharayil Gopalan, the Kerala AF Registry Investigators, Narayanan Namboodiri, A Jabir, A George Koshy, Geevar Zachariah, M Shifas Babu, K Venugopal, Eapen Punnose, K. U Natarajan, Johny Joseph, C Ashokan Nambiar, P.B Jayagopal, P.P Mohanan, Raju George, Govindan Unni, C. G Sajeev, N Syam, Anil Roby, Rachel Daniel, V.V Krishnakumar, Anand M. Pillai, Stigi Joseph, G.K Mini, Shaffi Fazaludeen Koya, Koshy Eapen, Raghu Ram, Cibu Mathew, Ali Faizal, Biju Issac, Sujay Renga, Jaideep Menon, D. Harikrishna, K. Suresh, Tiny Nair, S. S. Susanth, R. Anil Kumar, T. P. Abilash, P. Sreekala, E. Rajeev, Arun Raj, Ramdas Naik, Anoop Gopinath, R. Binu, Jossy Chacko, P T. Iqbal, N M Sudhir, Madhu Sreedharan, N. Balakrishnan, Muhammed Musthaffa, B. Jayakumar, Sheeba George, Anand Kumar, Thomas Mathew, V. K. Pramod, Muhammed Shaloob, Madhu Paulose Chandy, K. R Vinod, Karuana Das, Z. Sajan Ahamad, Pramod Mathew

**Affiliations:** ^1^ Liverpool Centre for Cardiovascular Science at University of Liverpool, Liverpool John Moores University, and Liverpool Heart and Chest Hospital Liverpool UK; ^2^ Department of Clinical Research Ananthapuri Hospitals and Research Institute Thiruvananthapuram India; ^3^ Department of Research Global Institute of Public Health Trivandrum India; ^4^ Cosmopolitan Hospital Trivandrum Kerala India; ^5^ Mother Hospital Thrissur Kerala India; ^6^ Amrita Institute of Medical Sciences Kochi Kerala India; ^7^ Danish Centre for Health Services Research, Department of Clinical Medicine Aalborg University Aalborg Denmark; ^8^ Department of Cardiology Ananthapuri Hospitals and Research Institute Thiruvananthapuram India

**Keywords:** atrial fibrillation, India, Kerala, sex differences, South Asia

## Abstract

**Background:**

Much data informing sex differences in atrial fibrillation (AF) comes from Western cohorts. In this analysis, we describe sex differences in Kerala, India, using the Kerala‐AF registry—the largest AF registry from the Indian subcontinent.

**Methods:**

Patients aged ≥18 years were recruited from 53 hospitals across Kerala. Patients were compared for demographics, treatments, and 12‐month outcomes, including major adverse cardiovascular events (MACE) and bleeding.

**Results:**

Male patients were more likely to have a smoking and/or alcohol history and had more ischaemic heart disease (46.2% vs. 25.5%; *p* < 0.001). Female patients had more valvular AF (35.1% vs. 18.0%; *p* < 0.001), and more use of calcium‐channel blockers (23.3% vs. 16.5%; *p* < 0.001) or digoxin (39.6% vs. 28.5%; *p* < 0.001). Almost one in four patients were not anticoagulated despite raised CHA_2_DS_2_‐VASc scores. 12‐month MACE outcomes did not differ by sex (male: 30.2% vs. female: 29.4%; *p* = 0.685), though bleeding events were more common in male patients (2.4% vs. 1.3%; *p* = −0.038), driven by minor bleeding (1.2% vs. 0.5%).

**Conclusion:**

In this large AF cohort from India, male patients had a higher prevalence of ischaemic heart disease, smoking, and alcohol use, while female patients had a higher prevalence of valvular heart disease. MACE did not differ by sex, though bleeding was more common in males. Almost a quarter of patients were not anticoagulated despite raised thromboembolic risk.

## Graphical abstract

This study describes the demographic, clinical, therapeutic, and 12‐month outcome differences between male and female patients in the Kerala‐AF registry. AF—Atrial Fibrillation. *Missing anticoagulation is defined as the absence of anticoagulation therapy despite an elevated CHA_2_DS_2_‐VASc score.

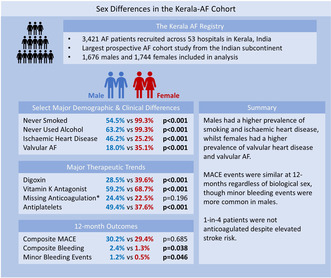

## 1 INTRODUCTION

Women with atrial fibrillation (AF) may have a higher risk of stroke, worse quality of life, and may be less frequently offered rhythm control therapy and anticoagulation therapies.^1^ However, much of the data informing these associations comes from Western cohorts. South Asian patients may have different risk factor profiles and different thromboembolic and bleeding risks compared to Western cohorts,^2^, ^3^ due in part to genetics and sociocultural effects—thus whether these sex differences hold true in unclear.

The Kerala‐AF registry^4^ provides data from over 3000 Indian patients from the Kerala region, which has recently undergone an epidemiological transition and is one of the most developed regions of India. This region now has a high prevalence of cardiovascular disease.^5^ Understanding differences between sexes could help inform future treatment of AF within this rapidly developing area and allow healthcare provision to be more precise and individualized. In this analysis from the Kerala‐AF registry, we report demographic, therapeutic, and 12‐month outcome differences between male and female patients with AF.

## 2 METHODS

### 2.1 Study design and cohort

The design of the Kerala‐AF registry has been described previously.^4^ Briefly, patients aged ≥18 years with documented AF were recruited between April 2016 and April 2017 across 53 hospitals in Kerala, India. Patients were recruited during attendance or admission to a recruiting hospital. Patients with potentially reversible causes of AF (e.g., as a result of myocardial infarction, sepsis, or postoperative AF) were excluded, as were critically ill patients with life expectancy <30 days. A case report form was used to capture medical history, examination, laboratory results, and imaging findings, obtained from review of clinical records and discussion with patients.

The dataset for this study has been previously described.^6^ The study was conducted in accordance with the Indian Council of Medical Research guidelines, and the ethical principles of the Declaration of Helsinki. All participants provided informed consent and only de‐identified data were shared for analysis.

### 2.2 Study endpoints

The primary clinical outcome in this analysis was a composite of major adverse cardiac events (MACE)—defined as all‐cause mortality, cerebrovascular accident (CVA), transient ischaemic attack (TIA), systemic embolism (SE), acute coronary syndrome (ACS) or hospitalization because of heart failure or arrhythmia—over 12‐month follow‐up. Secondary endpoints included composite bleeding events, comprising gastrointestinal (GI) bleeds, intracranial haemorrhage (ICH) and other minor bleeding events. Minor bleeding was defined as “any sign or symptom of hemorrhage (e.g., more bleeding than would be expected for a clinical circumstance, including bleeding found by imaging alone) that does not fit the criteria for major bleeding, but does meet at least one of: (a) requiring medical intervention by a healthcare professional; (b) leading to hospitalization or increased level of care; (c) prompting a doctor's consultation.” Comparisons were stratified by self‐reported biological sex.

### 2.3 Statistical analysis

Continuous variables were described as mean ± standard deviation and compared using *t*‐tests. As all continuous variables were reasonably normally distributed, medians and non‐parametric tests were not applied. Categorical data were described using counts and/or percentages and compared using two‐sample proportion tests, Chi‐square tests or Fisher's exact tests. Post hoc correction was not applied.

Demographics and therapeutic differences were described for the full cohorts. For clinical outcomes, as loss to follow‐up was considerable, we applied an algorithm to determine which patients to include in each outcome analysis, as follows: (a) if the patient had the event of interest, regardless of follow‐up duration, they were included; (b) if the patient completed 12‐month follow‐up without having the event, they were included; (c) if the patient died during follow‐up, they were included; (d) otherwise they were excluded. This approach ensured that patients with a known outcome, despite loss to follow‐up, could be included in analysis. Sensitivity analyses to assess the impact of loss to follow‐up were performed to ensure the robustness of findings; (a) assuming all lost patients remained event‐free, and (b) assuming all lost patients experienced the event.

As previously reported,^6^ missing data were handled either by removal of the variable, simple imputation, or multivariable imputation by chained equations (MICE), and one patient with apparent considerable data entry errors was excluded.

As statistically adjusted outcomes were previously described,^6^ demographic, therapeutic, and clinical outcome differences between male and female groups were described without adjustment, so as to describe real‐world trends and disparities. *p*‐values <0.05 were considered statistically significant. Statistical analysis was performed in R.

## 3 RESULTS

The full dataset included 3420 patients after the removal of one entry for suspected data entry errors. Of these, 1676 (49%) were male and 1744 (51%) were female.

### 3.1 Demographic differences

Demographic differences between male and female groups are shown in Table 1. Males were slightly older on average (65.4 ± 12.7 years vs. 63.9 ± 13.2 years; *p* = 0.001). There were significant disparities in smoking rates, with over 99% of females never having smoked as compared to 54.5% of males (*p* < 0.001)—though only 5.8% of males reported current smoking. Similarly, over 99% of females reported no history of alcohol use, compared with 63.2% of males (*p* < 0.001), while 27.7% of males reported current use.

**TABLE 1:** Demographic differences between male and female cohorts.

	Male (*n* = 1676)	Female (*n* = 1744)	*p*
Age (mean ± SD)	65.4 ± 12.7	63.9 ± 13.2	**0.001**
BMI (mean ± SD)	24.3 ± 3.5	24.3 ± 4.1	0.998
Smoking Status (*n*, %)			**<0.001**
Current	97 (5.8)	2 (0.1)	
Past	665 (39.7)	10 (0.6)	
Never	914 (54.5)	1732 (99.3)	
Alcohol Use (*n*, %)			**<0.001**
Current	465 (27.7)	7 (0.4)	
Past	152 (9.1)	5 (0.3)	
Never	1059 (63.2)	1732 (99.3)	
Heart Failure (*n*, %)			**<0.001**
HFpEF	177 (10.6)	218 (12.5)	
HFrEF (LVEF <50%)	294 (17.5)	215 (12.3)	
None	1205 (71.9)	1311 (75.2)	
Co‐morbidities (*n*, %)			
Hypertension	936 (55.8)	904 (51.8)	**0.020**
Diabetes Mellitus	911 (36.5)	571 (32.7)	**0.023**
Dyslipidaemia	774 (46.2)	692 (39.7)	**<0.001**
Ischaemic Heart Disease	775 (46.2)	445 (25.5)	**<0.001**
Chronic Respiratory Disease	370 (22.1)	343 (19.7)	0.084
Renal Impairment*	769 (45.9)	877 (50.3)	**0.010**
Chronic Liver Disease	44 (2.6)	23 (1.3)	**0.006**
Prior CVA, TIA or SE	239 (14.3)	259 (14.9)	0.628
Prior Bleeding Event	145 (8.7)	111 (6.4)	**0.011**
Pacemaker Implant	80 (4.8)	69 (4.0)	0.276

*Note*: Bold underline highlights significant results. Renal impairment encompasses a reported diagnosis of CKD or creatinine clearance <60 mL/min.

Abbreviations: CVA, cerebrovascular accident; HFpEF, heart failure with preserved ejection fraction; HFrEF, heart failure with reduced ejection fraction; SD, standard deviation; SE, systemic embolism; TIA, transient ischaemic attack.

Overall rates of heart failure were similar, though significantly more males had heart failure with reduced ejection fraction (HFrEF; male: 17.5% vs. female: 12.3%; *p* < 0.001) and numerically more females had heart failure with preserved ejection fraction (HFpEF; male: 10.6% vs. female: 12.5%; *p* = 0.078).

Significant differences were also observed in cardiovascular co‐morbidities, with most being more common in males, though absolute differences were small in most cases (Table 1). A particularly large disparity was seen in ischaemic heart disease (IHD; male: 46.2% vs. female: 25.5%; *p* < 0.001). Renal impairment was more frequent in females (male: 45.9% vs. female: 50.3%; *p* = 0.011).

### 3.2 Clinical differences

Clinical differences between male and female patients are shown in Table 2. Female patients had a significantly higher rate of valvular AF (male: 18.0% vs. female: 35.1%; *p* < 0.001) and were more likely to be symptomatic (male: 83.4% vs. female: 87.8%; *p* < 0.001), though similar numbers reported higher (III–IV) grades on the New York Heart Association (NYHA) classification (male: 17.8% vs. female: 17.3%; *p* = 0.721). Males more often had atypical symptoms (defined as chest pain, dyspnoea, or fatigue based on prior studies^7^; male: 50.9% vs. female: 44.5%; *p* < 0.001).

**TABLE 2:** Clinical differences between male and female cohorts.

	Male (*n* = 1676)	Female (*n* = 1744)	*p*
Valvular AF (*n*, %)	302 (18.0)	612 (35.1)	<0.001
Symptomatic AF (*n*, %)	1398 (83.4)	1532 (87.8)	<0.001
Atypical Symptoms (*n*, %)^a^	853 (50.9)	776 (44.5)	<0.001
NYHA class III‐IV (*n*, %)	299 (17.8)	302 (17.3)	0.719
AF Classification (*n*, %)			<0.001
Paroxysmal	706 (42.1)	643 (36.9)	
Persistent	319 (19.0)	293 (16.8)	
Permanent	651 (38.8)	808 (46.3)	
CHA_2_DS_2_‐VASc Score	2.7 ± 1.7	3.3 ± 1.7	<0.001
Adjusted CHA_2_DS_2_‐VASc Score^b^	2.7 ± 1.7	2.3 ± 1.7	<0.001
HAS‐BLED Score	2.6 ± 1.5	2.1 ± 1.4	<0.001
LA Diameter (mm) (mean ± SD)	41.8 ± 7.6	41.8 ± 7.4	0.935
LV Ejection Fraction (mean ± SD)	54.9 ± 11.9	57.7 ± 10.5	<0.001
Reason for Index Consultation (*n*, %)			
AF	1167 (69.6)	1229 (70.5)	0.601
Coronary Disease	159 (9.5)	87 (5.0)	<0.001
Heart Failure	97 (5.8)	70 (4.0)	0.017
Hypertension	20 (1.2)	22 (1.3)	0.878
Other/Not Known	148 (8.8)	131 (7.5)	0.169
Stroke/TIA/SE	17 (1.0)	32 (1.8)	0.045
Valvular Disease	68 (4.1)	173 (9.9)	<0.001

Abbreviations: AAD, anti‐arrhythmic drug; AF, atrial fibrillation; CCB, calcium‐channel blocker; LA, left atrial; LV, left ventricular; SD, standard deviation; SE, systemic embolism; TIA, transient ischaemic attack.

^a^ Atypical symptoms were defined as chest pain, dyspnoea or fatigue, as used in prior studies.^7^

^b^ Adjusted score subtracts 1 point from female patients as all of this cohort has +1 point for biological sex.

Male patients had higher rates of paroxysmal AF (male: 42.1% vs. female: 36.9%) and persistent AF (male: 19.0% vs. female: 16.8%), whilst females more frequently had permanent AF (male: 38.8% vs. female 46.3%); overall (*p* < 0.001).

Mean CHA_2_DS_2_‐VASc scores were higher in females (male: 2.7 ± 1.7 vs. female: 3.3 ± 1.7; *p* < 0.001). As the female sex counts +1 point towards the score, we also compared an adjusted score subtracting 1 point from female patients—this was lower in females (male: 2.7 ± 1.7 vs. female: 2.3 ± 1.7; *p* < 0.001). Overall, given prior associations between female sex and stroke risk, females were at higher predicted thromboembolic risk, but this was driven by female sex itself, and risk‐increasing co‐morbidities were more prevalent in males. HAS‐BLED scores were also slightly lower in females (male: 2.6 ± 1.5 vs. female: 2.1 ± 1.4; *p* < 0.001).

Reasons for index hospitalization in male patients were more often for coronary disease (male: 9.5% vs. female: 5.0%; *p* < 0.001) and heart failure (male: 5.8% vs. 4.0%; *p* = 0.020), whilst valvular heart disease was more frequently the reason for females (male: 4.1% vs. female: 9.9%; *p* < 0.001).

### 3.3 Therapeutic differences

Therapeutic differences between male and female patients are shown in Table 3. Rhythm control was slightly more frequent in male patients but did not reach statistical significance (male: 17.1% vs. female: 14.9%; *p* = 0.077). Catheter ablation (male: 0.6% vs. female: 0.3%; *p* = 0.226) and left atrial appendage occlusion (LAAO; male: 0.1% vs. female: 0.4%; *p* = 0.202) were infrequently utilized in the cohort as a whole.

**TABLE 3:** Therapeutic differences between male and female cohorts.

	Male (*n* = 1676)	Female (*n* = 1744)	*p*
Rhythm Control Strategy (*n*, %)	287 (17.1)	259 (14.9)	0.076
Catheter Ablation (*n*, %)	10 (0.6)	5 (0.3)	0.201
LA Appendage Occlusion (*n*, %)	2 (0.1)	7 (0.4)	0.180
Medications (*n*, %)			
Beta‐blocker	991 (59.1)	937 (53.6)	0.001
Rate‐limiting CCB	276 (16.5)	405 (23.3)	<0.001
Digoxin	477 (28.5)	691 (39.6)	<0.001
Class I AAD	20 (1.2)	26 (1.5)	0.462
Class III AAD	353 (21.1)	319 (18.3)	0.043
Vitamin K Anticoagulant	993 (59.2)	1198 (68.7)	<0.001
Direct Oral Anticoagulant	117 (7.0)	74 (4.2)	0.001
Missing Anticoagulation^a^	409 (24.4)	392 (22.5)	0.196
Antiplatelet	828 (49.4)	655 (37.6)	<0.001

Abbreviations: AAD, anti‐arrhythmic drug; AF, atrial fibrillation; CCB, calcium‐channel blocker; LA, left atrial; LV, left ventricular; SD, standard deviation; SE, systemic embolism; TIA, transient ischaemic attack.

^a^ This represents the absence of anticoagulation despite a raised risk score (CHA_2_DS_2_‐VASc ≥2).

Beta‐blockers were more frequently used in males (male: 59.1% vs. female 53.6%; *p* = 0.001) whilst rate‐limiting calcium‐channel blockers (male: 16.5% vs. female: 23.3%; *p* < 0.001) and digoxin (male: 28.5% vs. 39.6%; *p* < 0.001) were more frequently used in females.

Direct oral anticoagulants (DOACs) were infrequently used but were more commonly prescribed for male patients (male: 7.0% vs. female: 4.2%; *p* = 0.001). Significantly more females were prescribed vitamin K anticoagulants (VKAs; male: 59.2% vs. female: 68.7%; *p* < 0.001). More males were prescribed antiplatelets (male: 49.4% vs. female: 37.6%; *p* < 0.001). A large proportion of the cohort were not anticoagulated despite a raised CHA_2_DS_2_‐VASc score (≥2), though this did not differ by sex (male: 24.4% vs. female 22.5%; *p* = 0.196).

### 3.4 Clinical outcomes

Twelve‐month clinical outcomes stratified by sex are shown in Table 4. 12‐month follow‐up was available for a similar percentage of males (73.4%) and females (77.0%); *p* = 0.597.

**TABLE 4:** 12‐month clinical outcomes in male and female cohorts.

	Male (*n* = 1676)	Female (*n* = 1744)	p
Composite MACE Outcome (%)	420 (30.2)	444 (29.4)	0.685
All‐cause Mortality	212 (15.5)	214 (14.4)	0.430
Thromboembolism	31 (2.3)	41 (2.8)	0.473
Acute Coronary Syndrome	102 (7.4)	94 (6.3)	0.267
Hospitalization^a^	102 (7.4)	130 (8.8)	0.218
Composite Bleeding Outcome (%)	33 (2.4)	20 (1.3)	0.038
GI Bleeding (%)	12 (0.9)	11 (0.7)	0.835
IC Bleeding (%)	5 (0.4)	2 (0.1)	0.271
Minor Bleeding (%)	17 (1.2)	8 (0.5)	0.046

*Note*: Note that total numbers of patients per outcome differ in this table—the reasons for this are described in the methods section.

Abbreviations: GI, gastrointestinal; IC, intracranial; MACE, major adverse cardiac events.

^a^ Hospitalization for heart failure or arrhythmia.

The composite MACE outcome occurred with similar frequency (male: 30.2% vs. female: 29.4%; *p* = 0.685), as did all‐cause mortality (male: 15.5% vs. female: 14.4%; *p* = 0.430). Of those who died, cardiac death was the most common cause and was numerically more frequent in males, but not to statistical significance (male: 78.3% vs. female: 71.5%; *p* = 0.118), whilst stroke‐related mortality was significantly more frequent in females (male: 9.4% vs. female: 17.8%; *p* = 0.016). Overall rates of thromboembolism, acute coronary syndrome (ACS), and hospitalization for heart failure or arrhythmia did not differ between the sexes.

The composite bleeding outcome was uncommon in both groups but occurred significantly more frequently in males (male: 2.4% vs. female: 1.3%; *p* = 0.038). This was mainly driven by minor bleeding events (male: 1.2% vs. female: 0.5%; *p* = 0.046), whilst gastrointestinal (GI) and intracranial (IC) bleeds did not significantly differ between cohorts.

### 3.5 Sensitivity analyses

Sensitivity analyses were performed for the composite MACE and bleeding outcomes to assess the impact of loss to follow‐up. The results are shown in the supplementary material. Table S1 demonstrates composite outcomes assuming all patients lost to follow‐up experienced the event, whilst Table S2 demonstrates composite outcomes assuming all patients lost to follow‐up did not experience an event.

In the primary analysis, the absolute difference in MACE events between male and female patients was 0.8% (*p* = 0.685). In sensitivity analysis, this varied between 0.4% (*p* = 0.813; Table S2) and 2.9% (*p* = 0.081; Table S1). In the primary analysis, the absolute difference in composite bleeding events between male and female patients was 0.7% (*p* = 0.046, with greater risk in males). In sensitivity analysis, this varied between 0.9% (*p* = 0.054; Table S2) and 4.4% (*p* < 0.001; Table S1).

In summary, overall trends from the primary analysis were relatively similar, with composite MACE events being similar between male and female patients, whilst bleeding events were more frequent in male patients.

## 4 DISCUSSION

In this analysis from the Kerala‐AF registry, we report observed differences between male and female patients. Our main findings were:

- Smoking, alcohol use, and cardiovascular co‐morbidities (especially IHD and HFrEF) were significantly more frequent in male patients; however renal impairment was more common in females.
- Valvular AF was significantly more common in females, as was permanent AF—and women were more likely to be treated with CCBs and/or digoxin.
- Women were more often symptomatic with AF and were considered to be at higher thromboembolic risk by CHA_2_DS_2_‐VASc score, though this was driven by the ‘female sex’ component of the score.
- Almost a quarter of the cohort with raised CHA_2_DS_2_‐VASc scores were not anticoagulated; use of DOACs was low in both sexes, but women were more frequently prescribed VKAs and males were more often prescribed antiplatelets.
- MACE outcomes occurred with similar frequency between the sexes, however, bleeding outcomes were more frequent in male patients.

Below, we discuss these findings in the context of the guideline‐recommended Atrial Fibrillation Better Care (‘ABC’) pathway.^8^

### 4.1 Avoiding stroke with anticoagulation

In our analysis, 12‐month composite MACE and thromboembolic outcomes did not differ between male and female patients. However, in this relatively sick cohort, all‐cause mortality was high, and—of those who died—stroke‐related death was significantly more frequent in female patients (male: 9.4% vs. female: 17.8%; *p* = 0.016).

Perhaps the most striking trend is that almost one in four patients were not anticoagulated despite raised CHA_2_DS_2_‐VASc scores. This emphasizes the need for education of patients and clinicians and improved access to medications. A 2020 survey suggested that access to preventative medications in Kerala may be limited by expense.^9^ Another survey in 2021 found that Indian clinicians tended to prefer VKAs over DOACs, primarily because of cost rather than lack of knowledge or familiarity.^10^ The lack of anticoagulation is perhaps even more stark given evidence that South Asian people may be more predisposed to thromboembolic disease because of higher levels of prothrombotic biomarkers (e.g., homocysteine and plasminogen activator inhibitor‐1).^11^ Furthermore, some studies show that South Asian people treated with VKAs have a lower time‐in‐therapeutic‐range compared with White people,^12^ which further emphasizes the importance of improving DOAC availability.

It is recognized that women are less likely to receive anticoagulation for AF on a global level.^1^ In our study, we did not observe a difference in overall anticoagulation between the sexes, however, women were more likely to be prescribed VKAs than men. This may be as a result of the higher rate of valvular AF seen in female patients in this cohort, as DOACs are not recommended in this setting.^13^ Rheumatic heart disease continues to be prevalent in India^14^ and efforts to improve this may reduce adverse outcomes, and indeed AF burden, in future generations. Whilst some studies have found no sex‐specific differences in the prevalence of rheumatic heart disease,^15^ others suggest this is indeed more prevalent in females.^16^ Additionally, the pattern of valvular involvement may differ between sexes, with females more likely to show mitral involvement,^16^ which defines the “valvular AF” phenotype. There are also potential genetic reasons why women with rheumatic heart disease may be predisposed to worse outcomes.^17^

Notably, we found that a raised predicted thromboembolic risk in females by the CHA_2_DS_2_‐VASc score, but this was driven entirely by female sex itself and indeed males had more co‐morbidities associated with increased risk. Recent data from Western cohorts suggest that the female sex may no longer be an independent predictor of thromboembolic risk,^18^ though our data suggest that mortality as a result of stroke, in the Kerala‐AF cohort, was higher in females.

A recognized risk with anticoagulation is bleeding, which was rarely reported in either group, but was more common in male patients. This may relate to the greater prevalence of cardiovascular risk factors—especially IHD—in males, resulting in more prescribing of antiplatelets (male: 49.4% vs. female: 37.6%; *p* < 0.001). This should be considered when reviewing such patients—as per international guidelines, antiplatelet monotherapy is not effective for thromboembolic risk reduction in AF, while combination therapy has limited supporting evidence outside of the setting of recent ACS or stent implantation.^13^

In those at high risk of bleeding, LAAO may be beneficial, however, this was performed in a very low numbers of patients in the Kerala‐AF cohort (*n* = 9), with no difference between sexes (Table 3).

### 4.2 Better symptom management with rate or rhythm control

On a global level, it is recognized that women are more likely to have atypical cardiac symptoms, are less frequently offered rhythm control, suffer more complications from catheter ablation, and potentially have worse outcomes as a result of these factors.^1^ Aside from implicit bias, which may result in later referrals and delayed initiation of rhythm control, potential reasons for disparities between sexes include higher left atrial pressure in women, differences in atrial myopathic processes and epicardial adipose tissue, and intrinsic electrophysiological differences including more non‐pulmonary vein triggers in female patients.^1^

Some studies suggest that rhythm control (especially catheter ablation) is more often used when “typical” symptoms are present^7^ but when symptoms are atypical or absent, women are less likely to receive rhythm control than men. Interestingly, in our study, atypical symptoms were more commonly observed in male patients (male: 50.9% vs. female: 44.5%; *p* < 0.001) and whilst rhythm control was more frequent in males, this did not reach statistical significance (male: 17.1% vs. female: 14.9%; *p* = 0.077).

Use of rhythm control in this cohort as a whole was low and was not independently associated with mortality benefit in prior analyses^6^—nonetheless, there are reasons to believe that prolonged AF is detrimental to cardiac and neurological function^19^ and that early rhythm control may be prognostically beneficial.^20^ Unfortunately, there is also evidence that female patients tend to have more side effects from antiarrhythmics and glean less benefit with higher complication risk from catheter ablation.^1^ These aspects require further study to understand and mitigate the underlying causes. In addition, much of the data supporting these findings comes from Western cohorts, so their applicability to South Asian cohorts is less clear. Further investigation into these trends to determine the underlying reasons for therapeutic differences would be of benefit.

In terms of rate control, beta‐blockers were more commonly prescribed for male patients, perhaps because of a slightly greater prevalence of HFrEF. It should be noted that there is conflicting evidence on whether beta‐blockers confer prognostic benefit in AF with heart failure.^21^, ^22^

Prior studies have shown digoxin is more frequently used in women, in keeping with our findings. Concerningly, Digoxin may be associated with a higher risk of mortality and of certain types of cancer,^23^, ^24^ especially oestrogen‐receptor‐positive breast cancer, suggesting use in women should be minimised where possible.

### 4.3 Cardiovascular risk factors

India is seeing an epidemic of cardiovascular disease, as well as risk factors such as hypertension, obesity, diabetes, and dyslipidemia, emphasizing the importance of healthcare, education, and preventative treatment.^3^ Smoking rates seen in our study fit with known Indian trends—being substantially more common in men, with prior estimates around 23% versus 3% of women.^25^, ^26^

BMI was similar between the sexes in our study. A similar trend was seen in a study of patients presenting with acute coronary syndrome in Kerala,^27^ though another study focused on diabetic patients found females were more frequently obese.^28^ Although mean BMI was in the normal range in our study, it was towards the upper limit in both sexes and some studies suggest that a lower normal range should be applied in South Asian people (i.e., classifying ≥23 as overweight, rather than ≥25) as a result of the prevalence of “normal weight obesity”—with a visually normal body habitus hiding excessive visceral adiposity—often seen in this population.^29^ For this reason, diet and physical exercise are important factors to address, though some social determinants of health are rooted in culture and religion, making them difficult to change. South Asian diets may be higher in sodium, sugar, and trans fats than recommended^30^ and Indian people may undertake less physical exercise than other demographics.^31^, ^32^ Social determinants are important—cultural elements and traditional household roles may lead to neglecting one's own health, particularly for South Asian women. Spare time is sometimes culturally viewed as time that should be spent helping the family, rather than investing in one's own health,^3^ and surveys have shown that South Asian women may defer health‐related decision making to their husbands.^3^ A 2016 study found poor overall levels of physical activity amongst Kerala residents, particularly women, which the authors hypothesised may relate to restrictions on women participating in outdoor sports.^33^

Education is important, as many people underestimate the impact of co‐morbidities and benefits of lifestyle modification.^34^ Similarly, studies suggest that medication adherence may be poor amongst South Asian people,^3^, ^35^ which may compound the aforementioned problems with medication availability and cost.

Overall, 12‐month MACE events did not differ significantly between sexes in this study. This is despite higher CHA_2_DS_2_‐VASc scores in females. This exposes a weakness of this scoring system—while it is simple to calculate and implement, all components are given equal weight. Picture, for example, a 65‐year‐old woman with no other risk factors and compare her to a 60‐year‐old man with poorly controlled hypertension and diabetes mellitus—the latter would clearly be at greater cardiovascular risk, but both would score 2 on CHA_2_DS_2_‐VASc. This underscores the need for clinical judgement when applying risk scoring systems. Machine learning methods may prove beneficial in overcoming these limitations.^36^

## 5 LIMITATIONS

This study reports data from a large cohort from Kerala. There was no randomization to any particular treatment as the registry is observational in nature. Our findings may not be generalizable to other areas of India or South Asia, nor other global cohorts, and may not apply to individuals born in Kerala who emigrate elsewhere. Our findings are also limited to 12‐month follow‐up. Furthermore, our cohorts were recruited from hospital admissions and thus may represent a ‘sicker’ cohort than the general population.

Discrepancies identified in the original dataset meant that an algorithm needed to be applied to determine which treatment strategy (rate vs. rhythm control) was utilized in each case, as previously reported.^6^ While we feel this was adequate to facilitate analysis, we cannot exclude inaccuracies. Also, as a large number of comparisons were made between groups, type 1 error (significance by chance) may have arisen in some cases.

## 6 CONCLUSION

In this analysis from the Kerala‐AF registry, male patients had a higher prevalence of ischaemic heart disease and smoking, whilst valvular AF was significantly more common in female patients. MACE events did not differ between the sexes, though males were more likely to suffer bleeding events, potentially as they more often took antiplatelets. One in four patients were not anticoagulated despite elevated risk scores, underlining the need for improvement in medication availability and education.

## FUNDING INFORMATION

The Kerala‐AF registry was supported by the Kerala Chapter of Cardiological Society of India through a one‐time research grant No. CSI/IEC/2017. The funder did not play a role in the design or running of the study nor in the analysis of results. No funding was received toward the analysis and writing of this manuscript.

## CONFLICT OF INTEREST STATEMENT

GYHL reports: Consultant and speaker for BMS/Pfizer, Boehringer Ingelheim, Daiichi‐Sankyo, Anthos. No fees are received personally. GYHL is a National Institute for Health and Care Research (NIHR) Senior Investigator and co‐principal investigator of the AFFIRMO project on multimorbidity in AF, which has received funding from the European Union's Horizon 2020 research and innovation program under grant agreement No 899871. DG reports: Speaker for Boehringer Ingelheim, Biosense Webster, and Boston Scientific. Proctor for Abbott. Research Grants from Medtronic, Biosense Webster and Boston Scientific. The other authors report no conflicts of interest.

## ETHICS STATEMENT

The study was conducted in accordance with the Indian Council of Medical Research guidelines, and the ethical principles of the Declaration of Helsinki.

## PATIENT CONSENT STATEMENT

All participants provided informed consent and only de‐identified data were shared for analysis.

## CLINICAL TRIAL REGISTRATION

N/A.

## Supporting information


Data S1.

## Data Availability

Data may be made available upon reasonable request to the corresponding author.
