# Supplementary material for: Sex differences in atrial fibrillation in India: Insights from the Kerala‐AF registry
Source: J Arrhythm. 2024 Dec 1;41(1):e13195. doi: 10.1002/joa3.13195 (PMC11730713; doi:10.1002/joa3.13195)
Supplement: Supplementary file 1 — Data S1. [file JOA3-41-e13195-s001.docx]

# Supplementary Material

**Table S1 – 12-month clinical outcomes demonstrating maximum impact of loss to follow-up**

|  | Male  (n=1,676) | Female  (n=1,744) | p |
| --- | --- | --- | --- |
| Composite MACE Outcome (%) | 703 (41.9) | 680 (39.0) | 0.081 |
| Composite Bleeding Outcome (%) | 339 (20.2) | 276 (15.8) | **<0.001** |

For this sensitivity analysis, we assumed that all patients lost to follow-up did experience an event. †Hospitalisation for heart failure or arrhythmia. GI – Gastrointestinal; IC – Intracranial; MACE – Major Adverse Cardiac Events.

**Table S2 – 12-month clinical outcomes demonstrating minimum impact of loss to follow-up**

|  | Male  (n=1,676) | Female  (n=1,744) | p |
| --- | --- | --- | --- |
| Composite MACE Outcome (%) | 420 (25.1) | 444 (25.5) | 0.813 |
| Composite Bleeding Outcome (%) | 33 (2.0) | 20 (1.1) | 0.054 |

For this sensitivity analysis, we assumed that all patients lost to follow-up did not experience an event. †Hospitalisation for heart failure or arrhythmia. GI – Gastrointestinal; IC – Intracranial; MACE – Major Adverse Cardiac Events.
